# The Relationship of Mobile-Based Social Network Addiction and Family Communication Patterns, with Behavioral Problems in Secondary School Students: The Mediating Role of Emotional Self-Regulation

**DOI:** 10.11621/pir.2023.0404

**Published:** 2023-12-01

**Authors:** Mohadeseh Fasihi, Mohammad Rostami

**Affiliations:** a University of Kurdistan, Sanandaj, Iran

**Keywords:** mobile-based social network addiction, family communication patterns, behavioral problems, emotional self-regulation

## Abstract

**Background:**

New technologies offer endless possibilities for students and schools, but as the use of smartphones increases, psychological and behavioral problems may also increase.

**Objective:**

To investigate the relationship of mobile-based social network addiction and family communication patterns on the one hand, and behavioral problems in students on the other, with a focus on the mediating role of emotional self-regulation.

**Design:**

This study used a quantitative approach and a cross-sectional design. The participants were 384 students (female/male: 226/168; mean age: 16 ± 1.98) in secondary high schools in Tehran in the academic year 2022–2023. The students were selected using convenience sampling. The data were collected online using the Revised Family Communication Pattern Scale (1994), Mobile-Based Social Network Addiction Questionnaire (2016), Child Behavior Checklist — Adolescent Version (2001), and the Affective Style Questionnaire (2010). The data were analyzed using structural equation modeling with SPSS-25 and AMOS-24 statistical software.

**Results:**

The study showed that emotional self-regulation plays a mediating role in the relationship between mobile-based social network addiction and internalized and externalized behavioral problems (*P* ≤ .05). The indirect effect of conversation orientation on internalized and externalized behavioral problems mediated by emotional self-regulation was not significant, but the indirect effect of conformity orientation on internalized and externalized behavioral problems with the mediation of emotional self-regulation was significant (*P* ≤ .05).

**Conclusion:**

Based on the findings, it is suggested that school officials and parents should develop emotional self-regulation and communication skills in students and parenting skills in their parents to prevent and reduce potential harm such as internet addiction and behavioral problems in students.

## Introduction

Smartphone addiction has intensified in recent times with the emergence of social media and networks, as this type of addiction stems from a personal desire to communicate socially (Romero-Rodríguez et al., 2020). Social network addiction is a type of behavioral addiction that adversely affects users’ behavior and morals and leads to changes in adolescents’ habits, lifestyles, and communication (Romero-Rodríguez et al., 2022). For example, social networks were found to increase a user’s internet communication, distancing the user from their other obligations and making them reluctant to establish relationships with family and friends (Liu, Hu, & Qi, 2022). Social networks have expanded tremendously in recent years and people have easier access to these networks at any time and place through smartphones. Mobile-based social network addiction can be a risk factor for behavioral problems, especially in students (Liu et al., 2022).

Behavioral problems are abnormal behaviors that exceed the normal intensity and duration for a certain age. These problems can appear during developmental stages and can lead to serious behavioral defects and emotional problems (Yang et al., 2019). In addition to students, behavioral problems also have negative effects on their parents and teachers. These problems can be a negative predictor for students during their academic years and may turn into mental disorders in their adult lives (Gargano et al., 2023). One of the most widespread and widely accepted classifications of behavioral problems is the distinction between internalizing and externalizing problems, which has a lot of empirical support. *Internalizing problems* are individual in nature, meaning that the experience of disturbance occurs within the individual and involves problems such as depression, withdrawal, and various types of anxiety. *Externalizing problems* appear in adolescents’ external behaviors, and patterns that are formed in conflict with others and include problems such as aggression or attention deficit/hyperactivity disorder symptoms (Eirich et al., 2022).

Family is another factor that may influence mobile-based social network addiction (Crespo Ramos et al., 2022). The family can be the root cause of children’s behavioral problems through dysfunctional family patterns and relationships, and incorrect parenting practices (Mamsharifiet al., 2021). The family is a system whose members interact based on family communication patterns. The concept of family communication patterns refers to the way members communicate with each other and family interactions (Fitzpatrick & Ritchie, 1994). Family communication patterns involve either conversation or conformity orientations. In *conversation-oriented* families, members can freely express their opinions and attitudes. In these families, there is a high level of internal communication between the members (Campbell-Salome et al., 2019), and the children in such families are likely to experience better levels psychological well-being (Zarnaghash et al., 2013). In contrast, in *conformity-oriented* families, each member tries to force other members to follow their attitudes and beliefs. Children in these families learn to accept other people’s opinions unquestioningly, and thus they develop a low level of mental health (Koerner & Fitzpatrick, 1997). One study examined academic achievement and behavioral problems in primary and secondary school students and found that students who have close and intimate family relationships have fewer behavioral problems and, as a result, experience better academic adjustment (Smith et al., 2001).

One of the most essential skills that students must acquire for emotion and behavior control is emotion regulation (Graziano & Hart, 2016). Emotion regulation affects an individual’s performance in different situations and people try consciously or unconsciously to change their desires, behavior, and emotions in a positive direction (Gross & Jazaieri, 2014). A low level of emotion regulation is one of the reasons for excessive use of the internet (LaRose et al., 2003). Emotional self-regulation affects behavior and mental functioning, and people’s failure to regulate their emotions leads to emotional and behavioral problems. A recent study showed that the relationship between family functioning and social media addiction in adolescents is both directly and indirectly mediated by emotion regulation (Ghafoori & Haghayegh, 2021).

In recent years, especially due to the COVID-19 pandemic and the promotion of online education in most countries, special attention has been paid to the effect of smartphones, social networks, and mobile-based social network addiction on school staff and students. Studies have suggested that COVID-19 has adversely affected the quality of life of individuals and some feel extremely lonely, resorting to excessive use of the internet (Karakose et al., 2022a). Moreover, studies have shown that psychological distress induced by COVID-19 directly affects job burnout, depression, and addiction to social media (Karakose et al., 2022b). The decrease in students’ social interactions and the increase in the time they stay at home due to the COVID-19 pandemic have increased the use of online communication tools and, as a result, the risk of developing mobile-based social network addiction (Bruni et al., 2021).

Studies that have addressed the effects of COVID-19 on mobile-based social network addiction, as well as the consequences of mobile-based social network addiction and social networks and other related factors, including family communication patterns, on students’ subsequent behavioral and health problems, were either not necessarily focused on students or were not as comprehensive as the present study (Albursan et al., 2022; Erdner & Wright, 2018; Lu & Yeo, 2015; Satoorian et al., 2016; Smith et al, 2001). Furthermore, some of these studies failed to address the role of some variables (including emotional self-regulation) that can moderate and reduce the negative consequences of social network addiction and dysfunctional family communication patterns in students (Crespo Ramos et al., 2022; Mamsharifiet al., 2021). To bridge this research gap, the present study aimed to find out whether mobile-based social network addiction and family communication patterns are correlated with behavioral problems in students, using a comprehensive statistical model. The findings of this study can highlight the role of emotional self-regulation in reducing students’ behavioral problems as the goal of psychological and training interventions for school counselors in working with students with behavioral problems. The findings can also enhance knowledge about the variables in question.

### Research Hypotheses and Model

- There is a significant relationship between mobile-based social network addiction and students’ behavioral problems.
- There is a significant relationship between family communication patterns and students’ behavioral problems.
- There is a significant relationship between mobile-based social network addiction and students’ behavioral problems, with a mediating role of emotional self-regulation.
- There is a significant relationship between family communication patterns and students’ behavioral problems with a mediating role of emotional self-regulation.

## Methods

### Participants

The present study used a quantitative approach and a cross-sectional design. This fits the objectives of the study and seeks to quantify the participants’ responses and describe the observed reality. The research population consisted of all secondary high school students in Tehran in the academic year 2021–2022. According to Kline (2015), the number of participants in structural equation modeling (SEM) studies should not be less than 200. According to the available statistics, the total population of high school students in Tehran is 253,398 (Statistical Center of Iran [SCI], 2021). Using Morgan’s sampling table, the sample size was estimated as 384 students. Thus, taking into account the possibility of dropout, the initial sample size was considered to be 400 students, and that number of questionnaires were distributed to participants. However, after excluding incomplete questionnaires, the data from 384 students were used for analysis. The students were selected through convenience sampling due to the COVID-19 outbreak and inaccessibility of the students. A majority of participants were tenth-grade students (66.9%); 44% were studying experimental sciences; 46.4% were 16 years old; the majority were female (59%).

### Procedure

#### Questionnaires

##### Child Behavior Checklist (CBCL)

This instrument was developed by Achenbach (1991) to assess behavioral problems in children and adolescents aged 11 to 18. The checklist has 112 items that are scored on a 3-point Likert scale (0 *= absent,* 1 *= occurs sometimes, and 2 = occurs often*). The total score in CBCL ranges from 0 to 240. The checklist is one of the most widely used self-measurement tools for behavioral problems. In the original version, the researchers reported the test-retest reliability and internal consistency for behavioral problems to be .97 and .94, respectively; for externalizing behavioral problems as .94 and .92; and for internalizing behavioral problems as .91 and .90. A study in Iran reported Cronbach’s *alpha* to be .83 for the whole scale and .86 and .48 for the subscales of internalizing and externalizing problems, respectively (Minaei, 2007). In the present study, Cronbach’s *alpha* was .81 for the whole scale and .84 and .86 for the internalizing behavioral problems and externalizing behavioral problems subscales, respectively.

##### Revised Family Communication Patterns (RFCP)

The RFCP was developed by Fitzpatrick and Ritchie (1994) to assess conversation orientation and conformity orientation. This self-assessment scale has 26 items with five options ranging from *strongly agree* to *strongly disagree.* The first 11 items measure conformity orientation and the remaining 15 items measure conversation orientation. The main developers of the RFCP have reported acceptable content, criterion, and construct validity and reliability (Cronbach’s *alpha* and test-retest) for this instrument (Fitzpatrick & Ritchie, 1994). In Iran, a study reported that the reliability of this tool using Cronbach’s *alpha* and the corresponding values for conversation orientation and conformity orientation were .87 and .81, respectively (Koroshnia & Latifian, 2008). In the present study, Cronbach’s *alpha* for conversation orientation and conformity orientation was .89 and .84, respectively.

##### Social Network Addiction Questionnaire (SNAQ)

This questionnaire was developed in Iran to measure the degree of use of social networks (Khajeahmadi et al., 2017). The first version of the SNAQ was developed with 27 items and in the psychometric analysis, one item was removed to enhance the content validity of the scale. Following the exploratory factor analysis, the number of items on the questionnaire was reduced to 23. The scale used in the present study has 23 items that measure four factors: individual performance, time management, self-control, and social relations. The internal consistency of the SNAQ was confirmed with Cronbach’s *alpha* of .92. Overall, the scale has acceptable content, face, and criterion validity (Khajeahmadi et al., 2017). In the present study, Cronbach’s *alpha* was .89, confirming the reliability of the questionnaire.

##### Affective Style Questionnaire (ASQ)

The ASQ is a 20-item instrument developed by Hofmann and Kashdan (2010). The items are scored on a 5-point Likert scale from *very untrue of me* (1) to *very true of me* (5). The ASQ has three subscales (concealing, adjusting, and tolerating), each with 8, 7, and 5 items, respectively. All items are directly and positively scored. In Iran, a study reported Cronbach’s *alpha* values for the subscales of adjustment, concealing, and tolerance as .70, .75, and .50, and the total reliability as .81. Overall, the construct validity indices for the three subscales were acceptable (Karsheki, 2013). In the present study, Cronbach’s *alpha* was .86 for the whole scale and .75, .80, and .56 for the adjustment, concealing, and tolerance subscales.

After receiving the required permits from the university and the Department of Education of Tehran Province, the researcher contacted the intended schools and provided the principals with information about the objectives and significance of the study and the research procedure. Since this study was conducted during the COVID-19 pandemic and school closures, the online link of the instruments with some instructions about the completion of the questionnaires and ethical considerations were submitted to the students in the form of audio files and text messages, through the students’ education application (Shad; A government platform in Iran used in schools during the COVID-19 outbreak) and WhatsApp messenger. To comply with the ethical protocols, the students’ data were kept confidential and participation in the study was voluntary. Moreover, the students could ask any question about the completion of the questionnaires. Finally, the collected data were entered into the SPSS-25 and AMOS-24 software for statistical analysis.

### Data Analysis

The data were analyzed using descriptive statistics (standard deviation and mean) and inferential statistics (correlation analysis and SEM). Pearson’s correlation test was used to examine the relationship between research variables, and the SEM was used to examine the hypotheses related to the mediating model.

## Results

Pearson’s correlation test was used to test the research hypotheses and specify the correlations between the variables. Table 1 presents the descriptive statistics for the research variables and the correlations between them. The correlation analysis shows that individual performance (*r* = .39), time management (*r* = .39), self-control (*r* = .42), and social relations (*r* = .33) have positive significant correlations with externalizing behavioral problems. Similarly, individual performance (*r* = .42), time management (*r* = .39), self-control (*r* = .50), and social relations (*r* = .30) have positive and significant relationships with internalizing behavioral problems. Conversation orientation has a significant negative correlation with externalizing behavioral problems (*r* = –.38) and internalizing behavioral problems (*r* = –.45) but conformity orientation has a significant positive correlation with externalizing behavioral problems (*r* = .40) and internalizing behavioral problems (*r* = .41). The analysis of the components of ASQ indicated that adjustment has no significant relationship with externalizing behavioral problems (*r* = –.09) and internalizing behavioral problems (*r* = –.03), but the components of concealing (*r* = .25) and tolerance (*r* = .28) have a positive and significant relationship with externalizing behavioral problems. In addition, the subscales of concealing (*r* = .41) and tolerance (*r* = .35) have a positive and significant relationship with internalizing behavioral problems (see *Table 1*).

**Table 1:** Descriptive statistics and the correlation matrix for the research variables

	Variables	1	2	3	4	5	6	7	8	9	10	11
CBCL	Externalizing	1										
	Internalizing	.64**	1									
SNAQ	Individual Performance	.39**	.42**	1								
	Time Management	.39**	.39**	.73**	1							
	Self-Control	.42**	.50**	.60**	.60**	1						
	Social Relations	.33**	.30**	.30**	.47**	.50**	1					
RFCP	Conversation	–.38**	–.45**	–.33**	–.25**	–.35**	–.14**	1				
	Conformity	.40**	.41**	.27**	.22**	.18**	.16**	–.53**	1			
ASQ	Adjustment	–.09	–.03	–.09	–.09	–.09	–.00	.04	.03	1		
	Concealing	.25**	.41**	.20**	.17**	.19**	.11**	–.24**	.24**	.43**	1	
	Tolerance	.28**	.35**	.15**	.13*	.09	.11	–.15**	.25**	.25**	.46**	1
	M	92.00	167.46	33.52	20.96	13.60	12.10	41.51	33.46	18.46	21.43	13.12
	SD	6.79	17.66	6.93	5.39	3.58	3.37	15.66	9.99	4.34	4.93	3.34

Note. CBCL = Child Behavior Checklist. SNAQ = Social Network Addiction Questionnaire. RFCP = Revised Family Communication Patterns. ASQ = A# ective Style Questionnaire. M = Mean. SD = Standard Deviation. ** = P < .01; * = P < .05.

**Figure 1.**
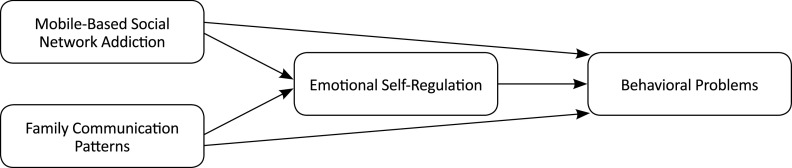
The hypothesized research model

SEM was run to find out whether the model for behavioral problems based on social network addiction and family communication patterns, with a focus on the mediating role of emotional self-regulation, fits the experimental data, as shown in Tables 2 to 4 and Figure 1. Table 2 shows the fit indices of the final model. Generally, each index obtained from AMOS software alone cannot confirm the fit or non-fit of the model, and these indices should be interpreted together. The values obtained for these indices confirm the goodness of fit indices of the model.

**Table 2:** Fit indices of path analysis of the adjusted model

Indices	Fit indices	
	Value	Acceptable range
X^2^/df	2.85	(Gaskin & Lim, 2016) < 3
RMSEA	.07	(Gaskin & Lim, 2016) < .1
CFI	.93	(Gaskin & Lim, 2016) > .9
NFI	.91	(Gaskin & Lim, 2016) > .9
GFI	.94	> .9

*Note. RMSEA = Root Mean Square Error Approximation.* *CFI = Comparative Fit Index. NFI= Normal Fit Index.* *GFI = Goodness of the Fit Index.*

**Table 3:** Indirect effect of family communication patterns and mobile-based social network addiction on behavioral problems mediated by emotional self-regulation

Criterion Variable	Predictor Variable	Type of Effect	B	Beta	Upper Bound	Lower Bound	Sig
	SNAQ	Indirect	.03	.03	.09	.00	.02
CBCL	Conversation	Indirect	–.13	–.03	.00	–.08	.08
	Conformity	Indirect	.36	.03	.13	.01	.04

*Note. CBCL = Child Behavior Checklist. SNAQ = Social Network Addiction Questionnaire. B = Unstandardized Coefficient. Beta = Standardized Coefficient. Sig = significance level.*

**Table 4:** Factor loads of the measurement models

	Variables	Standardized Weight	T	Sig
SNAQ	Individual Performance	.81	–	–
	Time Management	.84	17.11	.001
	Self-Control	.76	15.48	.001
	Social Relations	.52	10.15	.001
ASQ	Adjustment	.43	–	–
	Concealing	.96	6.26	.001
	Tolerance	.96	6.26	.001
CBCL	Externalizing	.75	–	–
	Internalizing	.86	13.94	.001
Conversation	Item 1	.49	10.38	.001
	Item 2	.70	16.52	.001
	Item 3	.78	19.32	.001
	Item 4	.74	17.81	.001
	Item 5	.64	14.47	.001
	Item 6	.74	17.93	.001
	Item 7	.78	19.40	.001
	Item 8	.84	22.00	.001
	Item 9	.81	20.51	.001
	Item 10	.80	19.19	.001
Conformity	Item 11	.80	20.12	.001
	Item 12	.68	15.61	.001
	Item 13	.79	19.85	.001
	Item 14	.82	20.89	.001
	Item 15	.86	–	–
	Item 16	.40	–	–
	Item 17	.49	6.54	.001
	Item 18	.69	7.54	.001
	Item 19	.78	7.81	.001
	Item 20	.66	7.42	.001
	Item 21	.54	6.87	.001
	Item 22	.47	6.38	.001
	Item 23	.87	8.05	.001
	Item 24	.68	7.05	.001
	Item 25	.58	7.08	.001
	Item 26	.58	7.08	.001

*Note. CBCL = Child Behavior Checklist. SNAQ = Social Network Addiction Questionnaire.* *ASQ = Affective Style Questionnaire. T = Critical Ratio. Sig = significance level.*

The data in Table 3 confirm the indirect effect of SNAQ on CBCL through ASQ at a 95% confidence interval (*P* ≤ .05). However, the data do not confirm the indirect effect of conversation orientation on CBCL through ASQ at a 95% confidence interval (*P* ≥ .05). In contrast, the findings confirm the indirect impact of conformity orientation on CBCL through ASQ at a 95% confidence interval (*P* ≤ .05). Table 4 and Figure 1 display the standardized path coefficients and the adjusted models.

As can be seen in Table 4, the factor loads for all four scales are significant at a 95% confidence interval (*P* ≤ .05).

**Figure 2.**
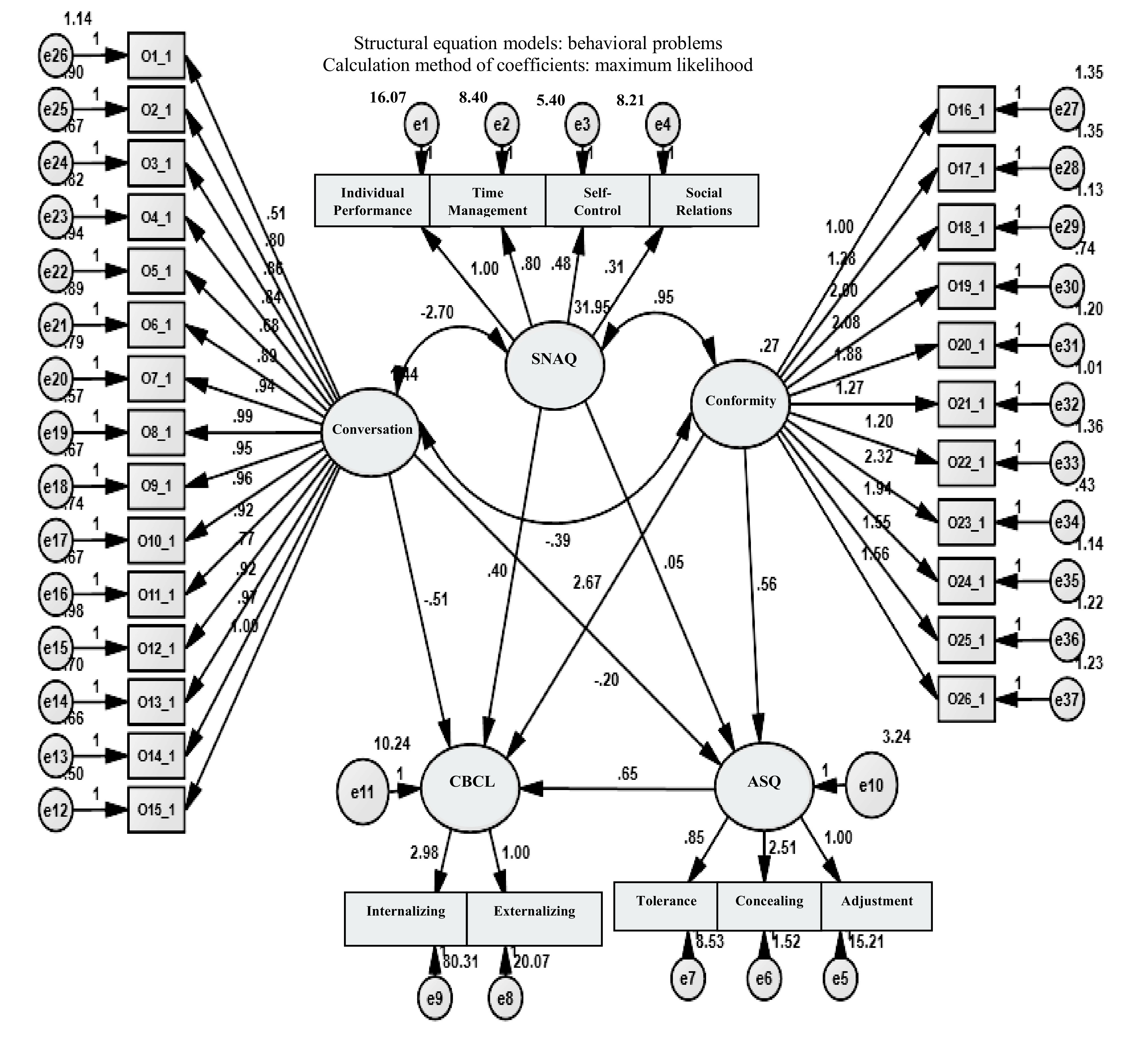
The adjusted model with standardized coe( cients

*Note. CBCL = Child Behavior Checklist. SNAQ = Social Network Addiction Questionnaire.*

*ASQ = Affective Style Questionnaire.*

## Discussion

The present study investigated the relationship between mobile-based social network addiction and family communication patterns on the one hand, and behavioral problems in secondary school students on the other, with a focus on the mediating role of emotional self-regulation. The results of the correlation analysis showed that individual performance, time management, self-control, and social relations have a positive and significant relationship with externalizing behavioral problems and internalizing behavioral problems, as confirmed in previous studies (Albursan et al., 2022; Elhai et al., 2016; Whang et al., 2003). Studies on users’ psychological characteristics have confirmed the relationship between depression and anxiety on the one hand, and problematic smartphone use on the other (Elhai et al., 2009). Other studies have found that internet addicts have a higher degree of loneliness and depression than non-addicts (Whang et al., 2003). Smartphone addiction was also associated with poor quality of life and academic procrastination in students (Albursan et al., 2022). These findings confirm a significant relationship between mobile-based social network addiction and behavioral problems in students.

The possible negative effects of the use of social networks on mental health and behavioral problems in users can be discussed from two perspectives: form and content. The form refers to excessive use of the internet, which leads students to distance themselves from most areas of real life. As a result, the students tend to experience symptoms of depression due to lack of sleep and feelings of loneliness, shame, and fatigue. Mobile-based social network addiction also causes a person to avoid social responsibility, isolate themselves, lose social support, become ineffective and perform poorly in school, all contributing to the exacerbation of behavioral problems in students (Sujarwoto et al., 2023). Two effective mechanisms can account for the impact of the content. The first is the cacophonous input (Ellison et al., 2007). Uncontrolled content that may be full of negative news, fake news, hoaxes, extortion, and cyber harassment leads to anxiety, frustration, and damage to mental health. Another mechanism is envy (Jordan et al., 2011). Social envy is a common feature of online social media. In social networks, students often compare themselves to others who may have a higher social and/or economic status. This psychological behavior may lead to decreased well-being as students feel inadequacy or failure in the online social community. This psychosocial mechanism is supported by the social rank theory of depression, which suggests that low self-concept is associated with depressive symptoms and suicidal risk (Sturman, 2011).

The results of correlation analysis in our study showed that conversation orientation has a negative and significant relationship with externalizing behavioral problems, but conformity orientation has a positive and significant relationship with externalizing behavioral problems. Moreover, conversation orientation has a negative and significant relationship with internalizing behavioral problems, while conformity orientation has a positive and significant relationship with internalizing behavioral problems. These findings are in line with previous studies (Crespo Ramos et al., 2022; Rueter & Koerner, 2008). Accordingly, we can argue that there is a significant relationship between family communication patterns and behavioral problems in students. One study showed that adopted adolescents in families with a more conformity orientation were significantly at a greater risk of adjustment problems (Rueter & Koerner, 2008). The authors stated that conversation orientation and conformity orientation determine the extent to which family members talk about and share their thoughts and feelings. Adolescents in conversation-oriented families who are more compatible with family members are approved by their parents and follow their parents’ demands, so behavioral problems are rarely seen in such families. However, people in families with conformity orientation have poor relationships. Instead of encouraging interpersonal skills and self-expression, they limit themselves to strict norms and a homogenous culture. Thus, behavioral problems will be more probable in such families (Fitzpatrick & Ritchie, 1994; Rueter & Koerner, 2008).

The data in the present study showed that emotional self-regulation plays a mediating role in the association between mobile-based social network addiction and behavioral problems, as evident in the literature (Mascia et al., 2020; Prado Gascó et al., 2018). These studies have demonstrated that people who can express and regulate emotions perform better psychologically and socially and have a high level of wellbeing (Prado Gascó et al., 2018). Bandura’s social cognitive theory (1986) defines self-regulation as self-observation and monitoring of feelings and behaviors. In accounting for the relationship between mobile-based social network addiction and behavioral problems, studies have focused on the suppression mechanism as one of the ineffective strategies that cause anxiety (Elhai et al., 2016). Individuals who continuously and addictively use smartphones tend to suppress negative emotions related to the real world, and as a result, by drowning in the internet, they show more weakness and anxiety in real relationships. By taking refuge in smartphones, they find an opportunity to regulate their suppressed emotions, and this vicious cycle constantly repeats itself (Hoffner & Lee, 2015). In contrast, individuals who reevaluate their emotions and behaviors have less problematic smartphone use and better real-life relationships (Elhai et al., 2016).

The findings of the present study also indicate that family communication patterns associated with conformity orientation have a significant and indirect impact on behavioral problems through emotional self-regulation, as reported by Xin et al. (2018). Lackova Rebicova et al. (2020) examined the relationship between adverse childhood experiences (ACEs) and emotional and behavioral problems and concluded that difficult communication or a complete lack of communication due to the absence of the mother and father increases the likelihood of emotional problems (Lackova Rebicova et al., 2020). Xin et al. (2018) showed that dysfunctional family communication patterns, including conformity orientation, are one of the risk factors for internet addiction. Indeed, adolescents with high self-regulation problems turn to online social networks more often due to their inability to establish social relationships in the real world. Furthermore, anxiety and emotional dysregulation cause adolescents to fail to establish effective relationships with other people. Thus, they try to compensate for their ineffective real relationships by taking refuge in mobile phones and joining online social networks, and as a result, they become dependent on and addicted to these networks and tend to conform to peers (Spratt et al., 2012).

## Conclusion

The results of the present study showed that emotional self-regulation plays a mediating role between mobile-based social network addiction and behavioral problems and between conformity orientation and behavioral problems. Emotional self-regulation is considered a factor effective in promoting students’ mental health and can help them avoid problematic behaviors through continuous review and control over their cognitive, emotional, and behavioral processes. While other studies (e.g., Crespo Ramos et al., 2022; Kim et al., 2009) on the negative consequences of social network addiction and dysfunctional family communication patterns have reported numerous findings, these studies have not necessarily introduced mechanisms that moderate and reduce these consequences and negative effects. The present study investigated the simultaneous effect of mobile-based social network addiction and family communication patterns on behavioral problems in students and showed that emotional self-regulation can be an effective mechanism in modulating and reducing behavioral problems in students. Thus, the present study provides new insights into the role of emotional self-regulation in moderating and reducing students’ behavioral problems. Given the gap in the literature, future research can provide more evidence for the role of emotional self-regulation in reducing behavioral problems that result from various emerging types of mobile-based social network addiction, including cyberloafing, computer game addiction, social network addiction, as well as interactive and negative parenting styles and patterns in families.

## Theoretical and Practical Implications

### Theoretical Implications

The findings of the present study concerning the relationship of mobile-based social network addiction and family communication patterns with behavioral problems in high school students, with a focus on the mediating role of students’ self-regulation, can be interpreted using Davis’s (2001) cognitive-behavioral model. According to this model, cognitive reasons and psycho-social health reasons can account for behavioral problems. While distorted cognitions (e.g., poor emotional self-regulation or low self-efficacy) cause many behavioral problems, poor psychosocial health (e.g., loneliness and interpersonal hostility) may be induced and worsened by mobile-based social network addiction and dysfunctional family communication patterns. They can also increase the individual’s vulnerability to behavioral problems and contribute to developing negative health consequences. According to this model, cognitive factors play a central role in behavioral problems. Thus, in line with the findings from the present study, students who suffer from behavioral problems due to mobile-based social network addiction or dysfunctional family communication patterns, such as conformity orientation, also have poor emotional self-regulation. Accordingly, promoting emotional self-regulation will probably lead to a decrease in behavioral problems in students (Yu et al., 2016). Empirical evidence supports the potential effects of cognitive and psychosocial health factors on mobile-based social network addiction (e.g., Kim et al., 2009; Wu et al., 2013).

### Practical Implications

First, school officials and counselors should consider the consequences of mobile-based social network addiction, as this type of addiction affects not only the students themselves, but also the academic performance and overall atmosphere of the school and all its students. Thus, school officials and counselors should hold workshops and preventive training programs for students. Such programs can contribute to strengthening social relationships (e.g., with teachers or classmates), and providing an environment where these students feel supported and valued. Second, self-regulation is a cognitive approach to controlling social network addiction and avoiding negative consequences related to it (Ho and Yang, 2018), and it can also contribute to reducing the negative effects of family communication patterns (Ghafoori & Haghayegh, 2021). Individual and group counseling sessions can be arranged at regular intervals to maintain students’ self-regulation. Research has shown that self-regulation plays an important role in achieving academic and personal goals, more than anything else (Gollwitzer & Sheeran, 2009). As a result, psychological and motivational training courses can help strengthen students’ self-regulation skills to deal with difficult situations.

## Limitations

This study has some limitations. First, the use of self-report instruments could affect the quality and validity of the participants’ answers. Second, the study was conducted based on cross-sectional data that might restrict causal inferences. Third, since the study was conducted during the COVID-19 pandemic, when schools were closed, the researcher was forced to distribute the questionnaires online. Accordingly, replicating similar studies with experimental and causal-comparative methods, especially by controlling the gender of students, can provide more reliable results.
